# Histomonosis in Poultry: A Comprehensive Review

**DOI:** 10.3389/fvets.2022.880738

**Published:** 2022-05-06

**Authors:** Lesleigh C. Beer, Victor M. Petrone-Garcia, B. Danielle Graham, Billy M. Hargis, Guillermo Tellez-Isaias, Christine N. Vuong

**Affiliations:** ^1^Department of Poultry Science, University of Arkansas Agricultural Experiment Station, Fayetteville, AR, United States; ^2^Facultad de Estudios Superiores Cuautitlan, Universidad Nacional Autonoma de Mexico, Cuautitlan Izcalli, Mexico

**Keywords:** *Histomonas meleagridis*, protozoa, parasite, turkey, histomonosis

## Abstract

*Histomonas meleagridis*, the etiological agent of histomonosis, is a poultry parasite primarily detrimental to turkeys. Characteristic lesions occur in the liver and ceca, with mortalities in turkey flocks often reaching 80–100%. Chickens and other gallinaceous birds can be susceptible but the disease was primarily considered sub-clinical until recent years. Treating and preventing *H. meleagridis* infection have become more difficult since 2015, when nitarsone was voluntarily removed from the market, leaving the poultry industry with no approved prophylactics, therapeutics, or vaccines to combat histomonosis. Phytogenic compounds evaluated for chemoprophylaxis of histomonosis have varied results with *in vitro* and *in vivo* experiments. Some recent research successes are encouraging for the pursuit of antihistomonal compounds derived from plants. Turkeys and chickens exhibit a level of resistance to re-infection when recovered from *H. meleagridis* infection, but no commercial vaccines are yet available, despite experimental successes. Safety and stability of live-attenuated isolates have been demonstrated; furthermore, highly efficacious protection has been conferred in experimental settings with administration of these isolates without harming performance. Taken together, these research advancements are encouraging for vaccine development, but further investigation is necessary to evaluate proper administration age, dose, and route. A summary of the published research is provided in this review.

## Introduction

The first known histomonosis outbreak was described by Cushman (1) and occurred in a Rhode Island turkey flock. Smith (2) further characterized histomonosis and attributed it to the protozoan *Amoeba meleagridis* obtained from liver lesions. Shortly thereafter, Tyzzer (3) more appropriately renamed this protozoon as *Histomonas meleagridis*. Further studies confirmed *H. meleagridis* as the etiological agent, although the mode of cecal invasion was still uncertain (4). Common synonyms for the disease have included blackhead disease, infectious enterohepatitis, histomoniasis, and typhlohepatitis (5–7). Blackhead disease is an unfortunate misnomer as a cyanotic head is neither pathognomonic nor common (8, 9); therefore, histomonosis will be the preferred terminology used throughout this review based on the Standardized Nomenclature of Animal Parasitic Diseases (10). Turkeys are especially susceptible to *H. meleagridis* infection, although other gallinaceous birds such as chickens, pheasants, and peafowls can be affected (7, 11, 12). Annual economic losses to the turkey industry have been estimated to exceed 2 million USD, and a 2020 survey listed histomonosis in position #11 of current issues facing the industry (9, 13).

**Figure 1:**
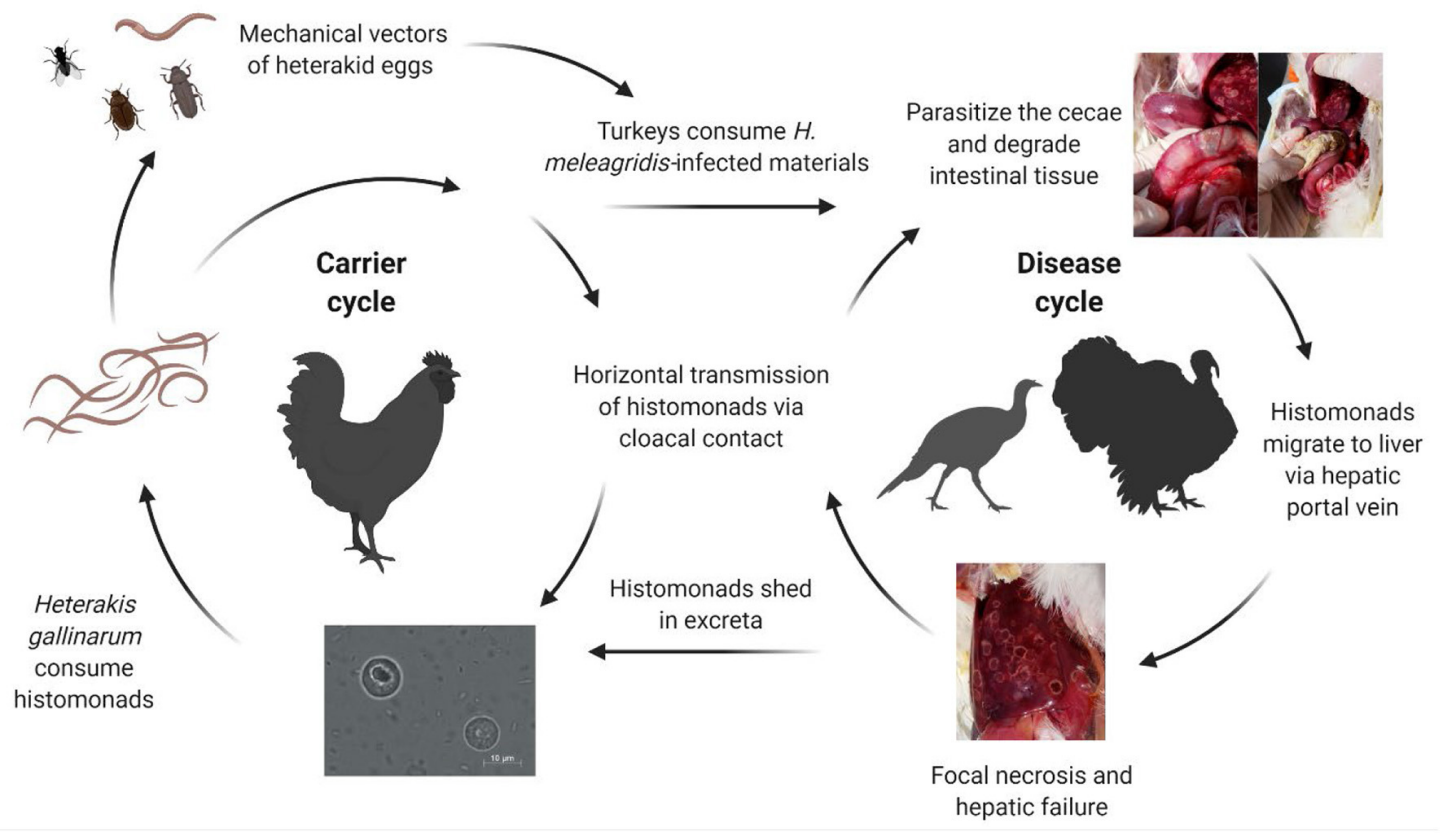
Complex transmission of *Histomonas meleagridis*. Histomonads can be consumed by *Heterakis gallinarum* and can be subsequently incorporated into the nematode ova. Carrier birds such as chickens can harbor the cecal worms and shed infected heterakid eggs into the environment. Earthworms, flies, and other invertebrates can serve as intermediate mechanical vectors of infected heterakid ova. Turkeys may ingest infected materials such as excreta or invertebrates contaminated with the protozoa. Once inside the intestine, the histomonads will migrate to the ceca, replicating and degrading the cecal lining. Direct transmission can occur rapidly from turkey-to-turkey due to cloacal drinking and reverse peristalsis movement of materials into the vent region (Created with BioRender.com).

Graybill and Smith (14) implicated *Heterakis* spp. in the role of transmitting *H. meleagridis* as they were unable to initiate the disease in absence of cecal worms. Further research showed that unprotected histomonads did not survive long periods outside the host, although duration in the environment when protected by feces or other materials was not well-characterized (15, 16). The separate rearing of poultry species is critical as chickens are considered partially resistant to histomonosis, frequently serving as asymptomatic carriers and reservoirs of *H. meleagridis*-infected heterakid eggs [Figure 1; (11, 17–21)]. Direct transmission within a flock is considered to occur through cloacal drinking which transfers materials from the vent region into the ceca through waves of reverse peristalsis (22–25). Horizontal transmission of *H. meleagridis* has occurred by comingling and contact of infected with uninfected turkeys, regardless of floor type and in absence of *H. gallinarum* (26, 27). The breed of turkeys or chickens may affect susceptibility to *H. meleagridis* infection, although male and female turkeys appear to be similarly susceptible; however, research is limited on the possible influence on disease development (28–31).

## Biology of *H. meleagridis*

**Figure 2:**
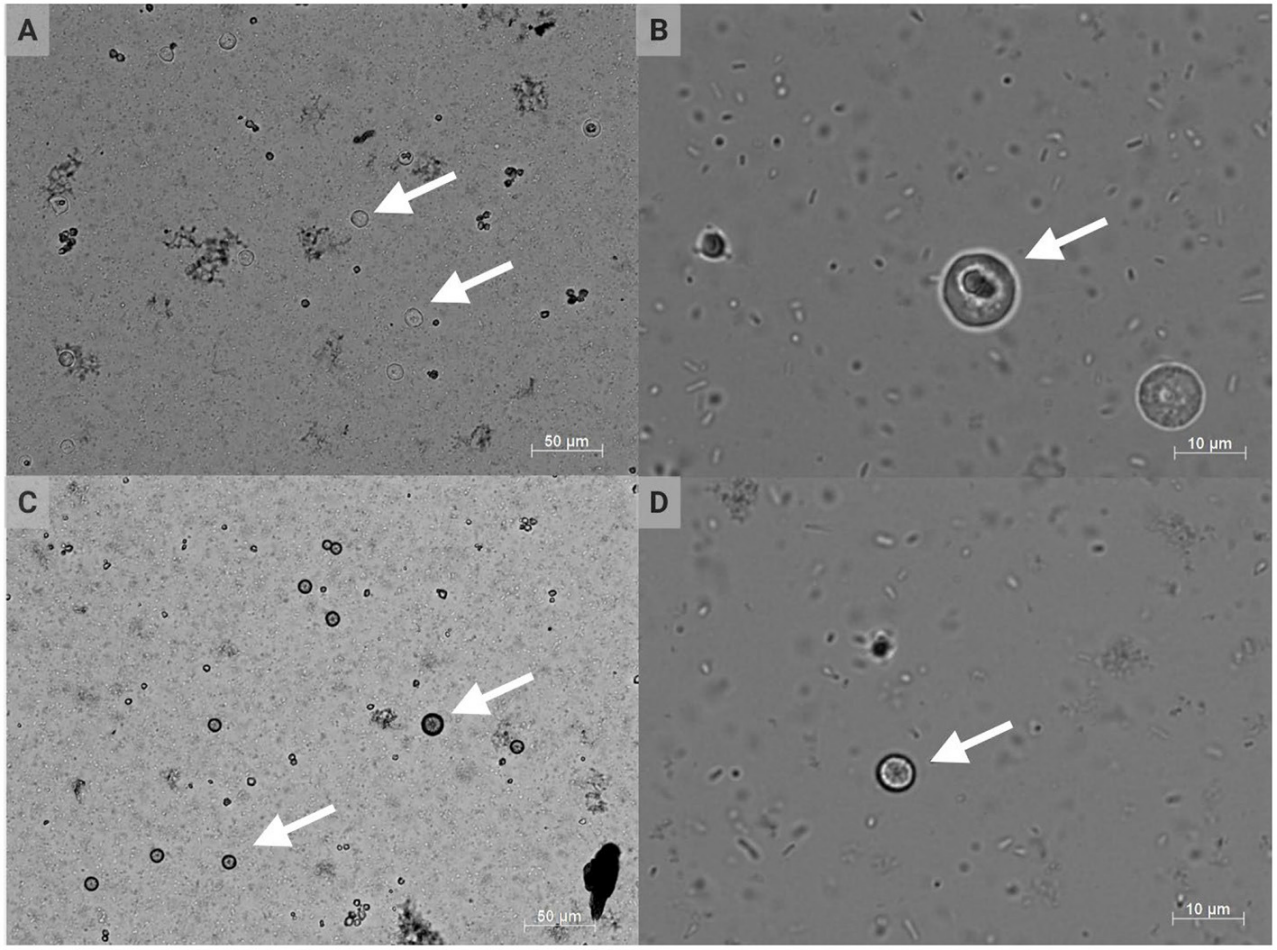
Conventional DIC photomicrographs of *Histomonas meleagridis*. Amoeboid or irregular morphology of histomonads at 200 × **(A)** and 1,000 × (**B**) total magnification; spherical morphology of histomonads at 200 × **(C)** and 1,000 × (**D**) total magnification. The arrows indicate selected histomonads (Created with BioRender.com).

**Figure 3:**
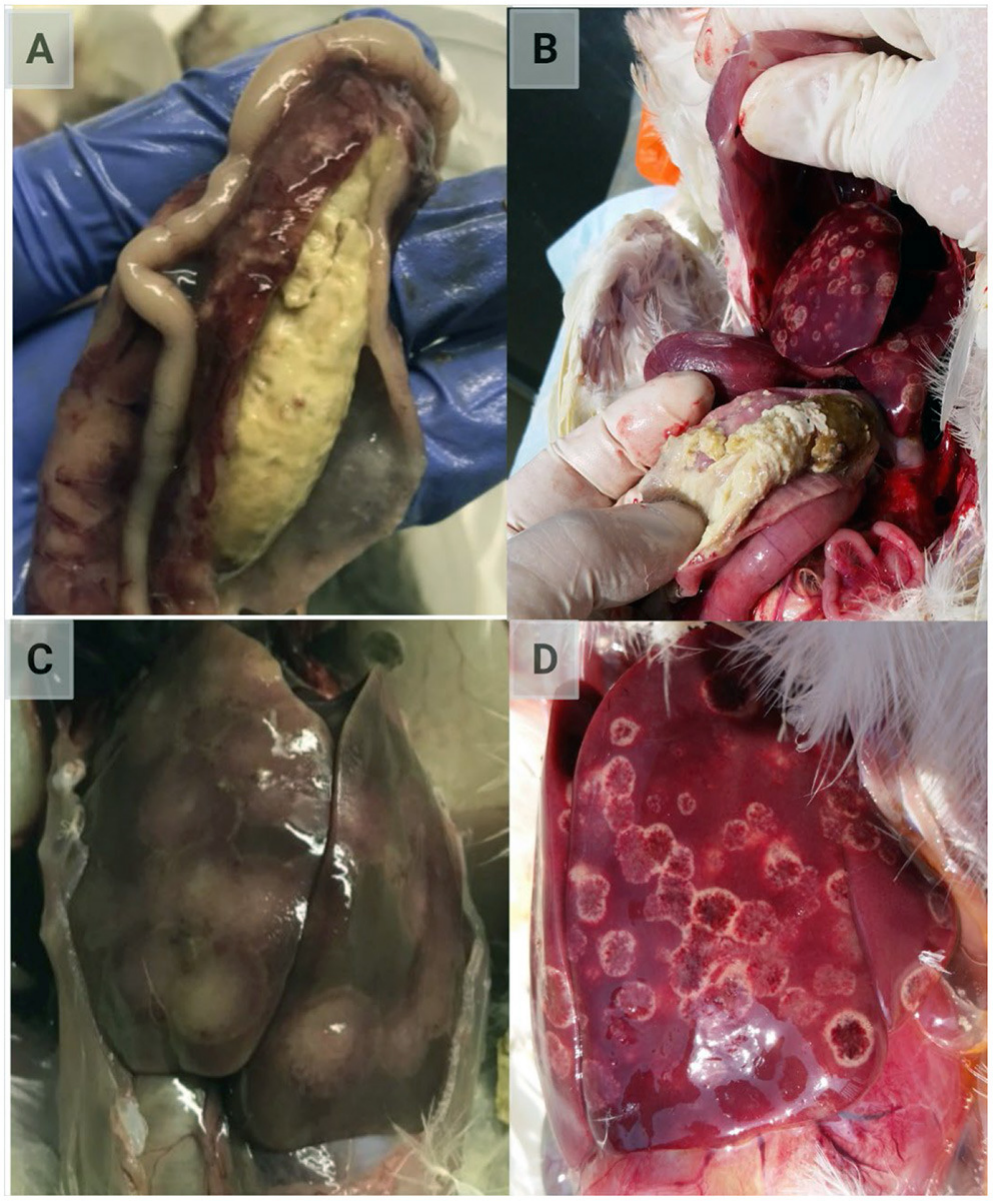
Classic lesions resulting from *Histomonas meleagridis* infection. **(A,B)** Caseous cheese-like cecal core; **(C,D)** focal necrosis resulting in target-like liver lesions (Created with BioRender.com).

*H. meleagridis* is a unicellular parasite belonging to the phylum Parabasalia, class Tritrichomonadea, order Tritrichomonadida, and family *Dientamoebidae* (32, 33). Interestingly, the morphology can change between flagellated and amoeboid forms depending on location within the ceca or liver, respectively, with an average histomonad size of 10–14 μm [Figure 2; (3, 5, 9, 34–36)]. The cell morphology and associated phenotypic changes have been mimicked experimentally *in vitro* (37). *H. meleagridis* typically exhibits a single-flagellated form within the cecal lumen, but this flagellum is lost upon mucosal invasion with the development of pseudopods (38). *H. wenrichi* (alternatively *Parahistomonas wenrichi*), a non-pathogenic but separate species, appears as 4-flagellated or amoeboid in form with a larger size of 20–30 μm (16, 39–41). *H. meleagridis* reproduce via binary fission; lacking mitochondria, these protozoa rely on hydrogenosomes as modified organelles for energy metabolism (3, 42–44).

Early *in vitro* work indicated that histomonads can be grown at temperatures of 36.5–37°C but not when reduced to 18–22°C for 48 h or 5°C for 24 h, suggesting that environmental survival of protozoa shed from infected birds is not likely to be culpable in mass infectivity (35). Currently, *in vitro* propagation occurs anaerobically at 40–41°C with a Medium 199-based cell culture and bacterial co-culture to simulate the body temperature and environment of a healthy turkey (44, 45). Dwyer's medium comprised of Medium 199, chick embryo extract, horse sera, and rice powder has been utilized, although other cell culture media such as L-15, MEM, or RPMI have been substituted effectively for Medium 199 (46). Modified Dwyer's medium, which removes the chick embryo extract and increases rice powder from 0.096 (w/v) to 0.8% (w/v), improved histomonad growth following revival of aliquots from liquid nitrogen and serial passage (47). Further increasing rice powder from 0.8 (w/v) to 4–8% (w/v) resulted in a nearly 10-fold growth increase, but this was not sustained longer than 2 days as the remaining nutrients became exhausted (48). Cholesterol supplementation has improved *H. meleagridis* growth *in vitro*, even in the absence of serum, which is typically required for adequate growth (49). Chute and Chute (50) cryogenically preserved *H. meleagridis* isolates in combination with 8% dimethylsulfoxide for up to 345 days and demonstrated viability of infection to birds following thaw. Honigberg and Dwyer (51) demonstrated that either 5 or 10% dimethylsulfoxide effectively preserved the protozoa in cryogenic storage as observed after 7 weeks; therefore, isolates could be maintained for future studies.

Field isolates of *H. meleagridis* can be cultivated from infected carcasses, particularly cecal samples, if shipment to a laboratory occurs soon after bird mortality with greater recovery potential if temperatures are maintained above 30°C (52). *In vitro* growth from cecal samples can usually be confirmed between 1 and 4 days after inoculation into culture media; intracloacal inoculation back into live birds can be used to further diagnose *H. meleagridis* as the original cause of infection in field outbreaks (53). Histomonads have also been isolated effectively from liver lesions, but *in vitro* propagation attempts in absence of bacteria have been unsuccessful (16, 34, 53, 54). Attempts to culture the protozoa in absence of live bacteria and serum were achieved with difficulty, but supplementation of palmitic acid or cholesterol was required along with antibiotic-killed bacteria and hamster liver extract; however, these results have not been easily replicated (45, 55–57). *In vitro* growth of *H. meleagridis* was better sustained with undefined populations of turkey cecal bacteria than with mixed chicken cecal bacteria (58). Moreover, histomonads have been grown with supplementation of single species of bacteria and monoxenic cultures have been established (59, 60).

## Pathogenesis

**Figure 4:**
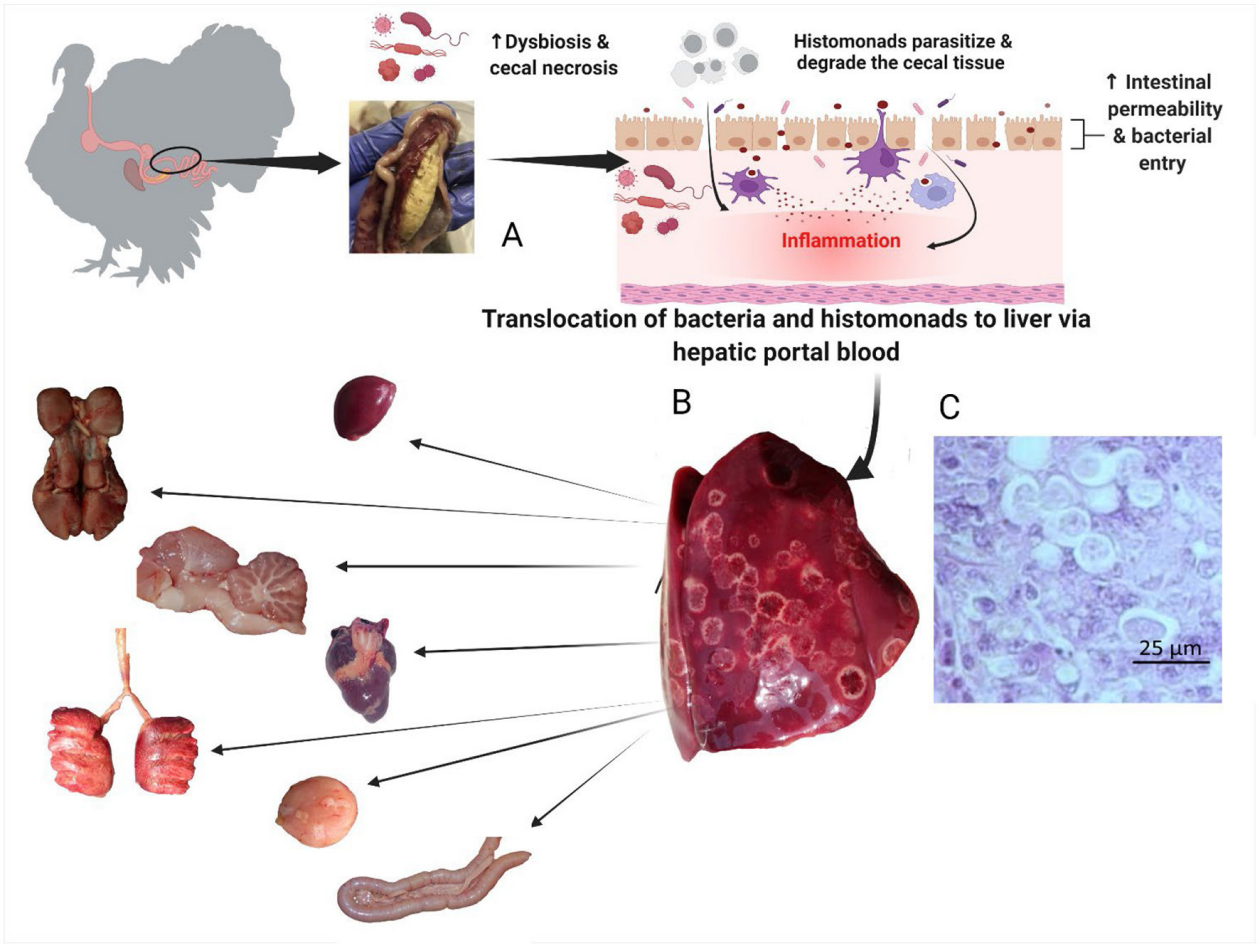
Pathogenesis of histomonosis. The parasite induces a severe inflammatory reaction in the ceca. The inflammatory reaction is followed by necrosis, with dysbiosis causing increased permeability in the ceca **(B)**. This allows bacterial and parasitic translocation to the liver via hepatic portal blood; the resulting pathognomonic lesions are exhibited as target-like liver lesions and caseous cecal cores **(A)**. *Histomonas meleagridis* in the liver of a turkey, Periodic acid–Schiff (PAS), 40 × **(C)**. From the liver, bacteria and histomonads migrate to other parenchymal organs (spleen, heart, kidneys, pancreas, lungs, brain, bursa of Fabricius) causing chronic systemic inflammation and multiple organ failure (Created with BioRender.com).

**Figure 5:**
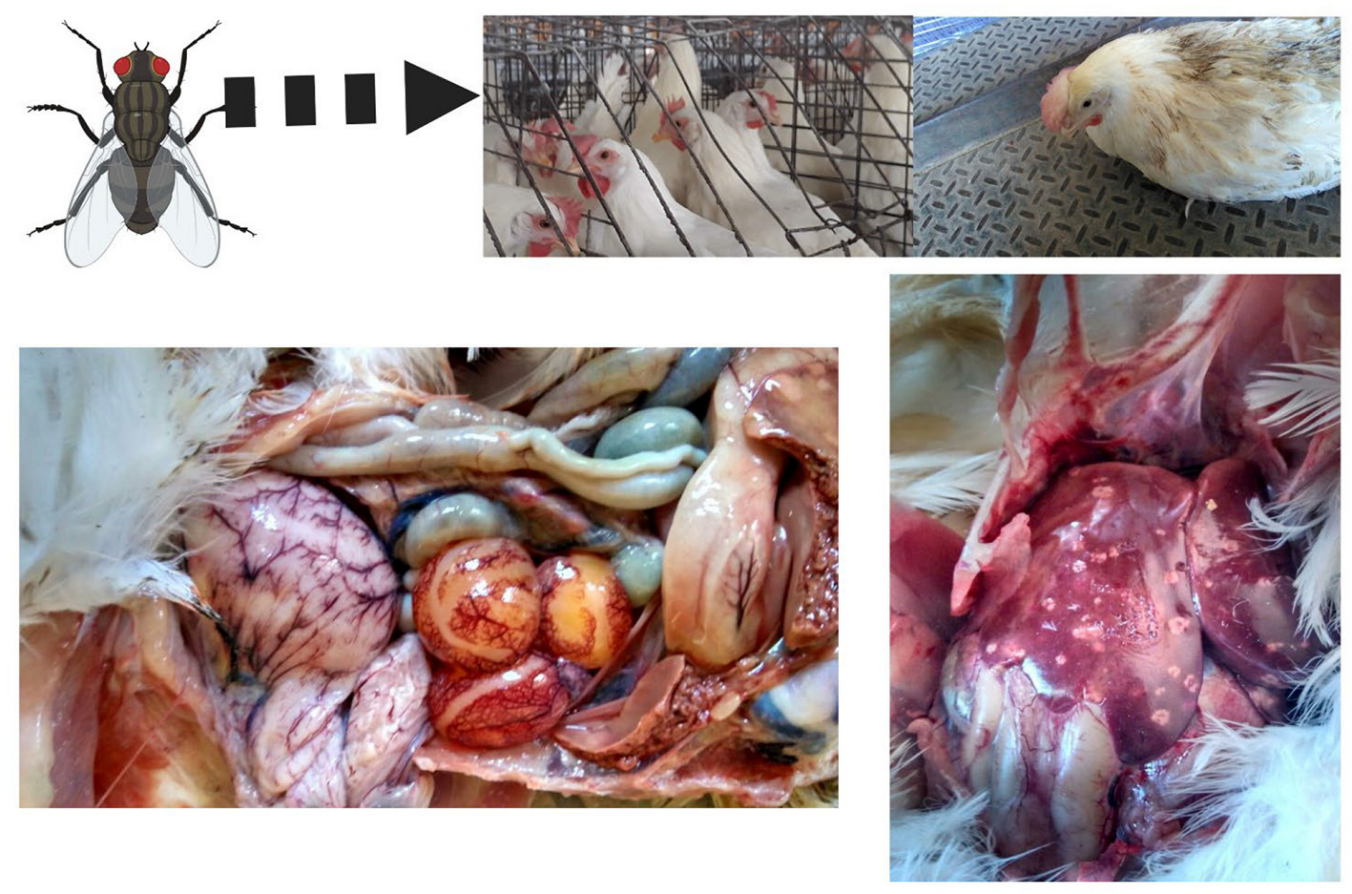
Field case of histomonosis in a layer operation. Turkeys are especially susceptible to *Histomonas meleagridis* infection, although other gallinaceous birds such as chickens, pheasants, and peafowls can be affected. In this figure, a cage layer hen in a commercial operation with multiple ages and a fly problem developed clinical signs and lesions of histomonosis without the presence of *Heterakis* spp. (Created with BioRender.com).

After parasitizing and degrading the cecal tissue, histomonads migrate to the liver via the hepatic portal blood; the resulting pathognomonic lesions are exhibited as target-like liver lesions and caseous cecal cores [Figures 3, 4; (44, 61, 62)]. Histomonads have been observed in the bursa of Fabricius of 6-week-old commercial chickens diagnosed with histomonosis, further implicating the intracloacal route for natural infection (63). Although less common, *H. meleagridis* has also been shown to infect areas including the brain, pancreas, heart, lungs, kidneys, and spleen (64–68). Turkeys are especially vulnerable to histomonosis, and chickens (Figure 5) are less susceptible but function to serve as reservoirs and can develop the disease (17). Cloacal transmission seems less important to chickens than turkeys for transfer of histomonosis, as horizontal transmission did not occur in the absence of vectors and was not exacerbated with *Eimeria adenoeides* challenge, which is not surprising as this *Eimeria* spp. is turkey-specific (69). While cloacal drinking is a well-known occurrence in chickens and turkeys, species differences in horizontal transmission could result from higher litter moisture and huddling behavior in turkeys than chickens, allowing greater survival and subsequent transmission of *H. meleagridis* in the absence of vectors (22, 69). Mortalities in turkey flocks can reach 80–100%; organic farms co-rearing turkeys and broilers have struggled with series of outbreaks with broiler and turkey mortalities reaching 100 and 67.2%, respectively, possibly due to co-infection with *Eimeria* spp. (19, 44, 70). Susceptibility of different poultry species and genetic lines has only been evaluated briefly, but infection incidence and severity do appear different (28, 29, 68, 71, 72). In chickens, sex-related variations and environmental differences have influenced intestinal structure and function; therefore, it seems reasonable that these differences could factor into the incidence and severity of histomonosis (73). In addition to age and genetic line of poultry, variations in mortality rate and lesion severity could result from strain-specific differences in virulence of *H. meleagridis* or exposure dose (23, 74, 75). Although chickens were previously regarded as sub-clinically affected by histomonosis, outbreaks have occurred recently in broiler breeder and free-range flocks (76, 77). Interestingly, recent research has indicated that *H. meleagridis* infection and replication are similar regardless of chicken genetic line, further suggesting that chickens may be asymptomatic or sub-clinically infected but not actually resistant to infection (78).

A virulent clonal strain of *H. meleagridis* induced similar mortality and pathology in turkeys regardless of age, sex, or dose (31). A low dose of 3,162 histomonads induced 100% mortality in British United turkeys (BUT-Big6) by 2-weeks post-infection (30). Three different genetic lines of turkeys showed similar susceptibility to histomonosis, although wild Canadian turkeys exhibited higher mortality rates and lower liver lesions than BUT-Big6 or Kelly-Bronze lines (72). Concurrent infection with *E. tenella* can aggravate the development of histomonosis in broiler chickens, specifically increasing liver lesions (79). Conversely, turkeys co-infected with *E. adenoeides* and *H. meleagridis* resulted in significantly reduced cases of histomonosis (24). The dosage and timing of *Eimeria* vaccination of chickens will influence the severity of aggravation due to histomonosis, although further co-infection studies are necessary to conclude effects of combined pathogens to severity in chickens and turkeys (80).

Bradley and Reid (81) inoculated gnotobiotic (bacteria-free) turkeys with *H. meleagridis* in combination with either *Escherichia coli, Bacillus subtilis*, or *Clostridium perfringens* and suggested that a combination of the protozoa and bacteria populations was required to initiate histomonosis. Incidence of *H. meleagridis* infection in gnotobiotic chickens and turkeys increased when concurrently challenged with a mixture of *E. coli* and *C. perfringens*, whereas histomonosis was lessened with administration of a single bacteria species (82). Healthy turkey ceca contain predominantly (>50%) anaerobic *Lactobacillus* spp. and relatively low (<1%) coliforms and *Enterococcus* spp. (61). *Salmonella typhimurium, E. coli*, and *H. meleagridis* infections have been found concurrently in broiler chicken flocks (83). Cultures of *H. meleagridis* were identified to favor obligate anaerobes of the *Clostridiaceae* family, aerotolerant anaerobes of the *Bacteriodaceae* family, or facultative to obligate anaerobes of the *Baccillaceae* family (84). The Proteobacteria phylum increased in relative abundance in birds with severe histomonosis, but *E. coli* populations were maintained at the same level in turkeys regardless of the level of gut inflammation (85). *E. coli* mutually benefited histomonad growth *in vitro* and increased cecal involvement *in vivo* (60, 86). Co-infection of laying chickens with *H. meleagridis* and *E. coli* produced severe dysbiosis, increased microscopic lesions, and enhanced colonization of the cecal tissue (86). Recently however, the gastrointestinal pathology and *E. coli* load were not associated with severity of histomonosis, while microbiota composition and dysbiosis were directly attributed to the severity of inflammation (85). In addition to providing direct nutrients, bacteria appear to serve a mutualistic role with the protozoa by supplying essential proteins and metabolites during replication, as well as regulating *in vitro* environmental conditions (87).

Histomonosis has been produced in experimental settings with the intracloacal inoculation of infected liver, cecal tissues, or with a suspension of *in vitro* cultivated *H. meleagridis* (15, 62). Variations in host resistance, challenge dose, pathogen virulence, and frequency of exposure are some factors influencing disease severity (88). A case reproductive rate of 8.4 was estimated in a horizontal transmission study and turkeys recovered from histomonosis were shown to remain infectious for 5.7 days after recovery (89). A retrospective data analysis implicated an increased relative risk of male commercial turkey grow-out flocks to contracting histomonosis when located within 1 mile of a broiler breeder flock (90). Lund (74) reported a positive correlation between infective dose (10^2^-10^5^ histomonads/birds) and mortality; conversely, a low dose of 10 histomonads induced 100% mortality in turkeys (91). Liebhart and Hess (92) administered a virulent isolate via oral administration to 1-day-old turkeys with successful initiation of histomonosis, but the oral route of infection remains controversial. Presumably, histomonads cannot survive the low pH in the ventriculus unless protected by a vector such as *Heterakis* spp. or with a neutral to alkaline pH in the gastrointestinal tract to allow survival of the protozoa (67, 93). *H. meleagridis* has been shown to persist up to 9 h in non-chlorinated water and fecal droppings and up to 6 h on materials such as feathers and feed (94). Histomonads are fragile when shed unprotected into the environment, but not much is known about the methods for disinfection (95). Consequently, the importance of *H. meleagridis*-infected water as a possible source of involvement for cloacal transmission has been suggested as an important risk factor (94, 96). Although previously disregarded to form resistant structures, cyst-like forms have recently been described *in vitro*, but the importance of these structures to pathogenesis is not yet understood (95, 97–100).

Oral challenge with virulent histomonads on day-of-hatch has previously induced histomonosis in turkeys (31), although the oral route in absence of vectors remains somewhat controversial in older birds. Recently, challenge with wild-type *H. meleagridis* before feeding on day-of-hatch induced disease regardless of oral or cloacal route, presumably due to the near-neutral pH in the proventriculus-ventriculus region allowing the histomonads to survive and parasitize the ceca (101). Interestingly, oral challenge with virulent *H. meleagridis* at day 21 did not induce histomonosis, further suggesting that the cloacal route rather than the oral route is the primary method for transmitting unprotected histomonads in older birds; however, the oral route should not be disregarded for young birds (101).

Tyzzer (102) indicated the survival of *H. meleagridis* within heterakid ova for 2 months during winter temperatures. Heterakids can thereby serve as primary transmitters for initial introduction of disease due to infected ova withstanding environmental conditions for long durations (18, 103–105). Histomonads are released when the infected *Heterakis* spp. larvae hatch in poultry (106, 107). Lifetime fecundity of *H. gallinarum* is regulated by both inverse density and density-dependent mechanisms (108). *Alphitobius diaperinus* (darkling beetle or lesser mealworm) function as environmental contaminants for accidental introduction of *H. meleagridis* into a flock rather than serving as a primary transmitter like *Heterakis* spp. (109). The importance of *A. diaperinus* as a reservoir is uncertain due to the persistence of *H. gallinarum* and *H. meleagridis* DNA within dead beetles and litter from depopulated houses even after long periods (109, 110). *Lumbricus* spp. (earthworms) are not required for completion of the heterakid larvae or histomonad life cycles, serving rather as paratenic hosts and mechanical vectors if consumed by poultry (12, 20, 21, 111).

## Phylogenetic and Molecular Characterizations

Indirect and blocking ELISAs have been developed for detection of *H. meleagridis* but have not yet been rigorously tested for specificity or cross-reactivity to other related protozoa commonly found in field isolates (112, 113). An indirect sandwich ELISA has been used successfully to identify *H. meleagridis* infections in pullet and layer flocks (114). Other parasites such as *Tetratrichomonas gallinarum* and *Blastocystis* spp. may be present in field outbreaks and potentially confused with *H. meleagridis* (42, 115). Polymerase chain reaction (PCR) has been successfully utilized to detect *H. meleagridis* in samples and infected birds, as well as to differentiate from *T. gallinarum* and *Blastocystis* spp. (89, 116–120). DNA presence does not necessarily indicate active infection; therefore, diagnosis of histomonosis is recommended to include microscopy to confirm presence of the protozoa (78, 110, 115). *H. meleagridis* conforms similarly to other trichomonad parasites in structure and division; close phylogenetic relationships to *D. fragilis* and *Tritrichomonas foetus* were identified based on gene sequencing analysis of β-tubulin and small subunit rRNA genes (40, 121–123). Analysis of 18S rRNA and internal transcribed spacer (ITS)-1 sequences has demonstrated a clear distinction between *H. meleagridis* isolates and other trichomonads such as *D. fragilis* (98). Genetic sequencing and phylogenetic analysis of 5.8S rRNA and the flanking ITS-1 and ITS-2 regions revealed marked genetic diversity of *H. meleagridis* isolates (33). Furthermore, combinations of data obtained from Nanopore and Illumina sequencing platforms resulted in the assembly of genome sequences exhibiting gene deletions and truncations for two phenotypically different *H. meleagridis* isolates, indicating a difference in attenuated and virulent strains (124).

Analysis of 18S rRNA, α-actinin1, and *rpb1* genetic loci revealed two different phylogenetic clusters of *H. meleagridis* isolates in Europe and further identified two genotypes; in contrast, a probed sequence and partial 18S rRNA have displayed genetic similarity of six purportedly different isolates (33, 125). Biological relevance and incidence of these two distinct genotypes have not yet been fully elucidated (19, 125); although Grafl et al. (126) described a field outbreak of male turkeys with *H. meleagridis* genotype 2 infection as having severe typhlitis with limited hepatic lesions. Using micromanipulation, clonal cultures of *H. meleagridis* and other protozoa have been established which enable researchers to better understand pathogenicity, morphology, and genetic differences between species (75). Mono-eukaryotic cultures have also been established from mixed field samples containing *H. meleagridis, T. gallinarum*, and *Blastocystis* spp., and these monocultures could potentially better mimic field strains as opposed to clonal cultures while removing the interference of other protozoa (127). Thirty-seven unique surface and intracellular antigens were identified through analysis of a cDNA library generated from a monoculture and screened against polyclonal anti-*H. meleagridis* rabbit sera (128). A cDNA library generated from a non-clonal culture resulted in the identification of 3,425 putative genes belonging to *H. meleagridis* (84). Hydrogenosome protein-coding sequences and three different α-actinin proteins (α-actinin1, α-actinin2, α-actinin3) were identified and shown to be immunogenic to turkeys and chickens (128, 129). Humoral immune response to *H. meleagridis* α-actinin1 and α-actinin3 was higher and induced sooner in specific-pathogen-free layer-type chickens as compared to meat-type chickens (68). Shotgun proteomics has been utilized to compare virulent and attenuated mono-eukaryotic monoxenic *H. meleagridis*; cysteine proteases were the predominant lytic molecules in the virulent exoproteome as compared to the attenuated isolate (130, 131). Mazumdar et al. (132) completed a *de novo* transcriptome sequencing study utilizing single-cell cloned virulent and attenuated isolates, demonstrating different gene families. Proteomic comparisons have detected expression differences including upregulation of stress response, peptidase, and metabolic proteins in a low-passaged virulent *H. meleagridis* isolate; whereas an attenuated strain had higher expression of cellular division proteins (133, 134).

## Chemotherapy and Prophylaxis

Tyzzer (135) tested several trivalent arsenicals (including arsenious acid, atoxyl, neoarsphenamine, and tryparsamide) as chemotherapeutics against histomonosis, but with inconsistent results. Pentavalent arsenicals such as nitarsone (4-nitrophenyl-arsonic acid; Histostat-50^TM^), carbasone (4-carbamylamino-phenylarsonic acid), and roxarsone (3-nitro-phenylarsonic acid) offered fewer toxicity concerns than the trivalent compounds for poultry but also exhibited a narrower chemotherapeutic index (16, 136). Carbasone was highly effective in prevention of a field isolate of *H. meleagridis* (136). Nitroimidazole compounds (including dimetridazole, metronidazole, ornidazole, and tinidazole) were effective *in vitro* at concentrations of ≥10 μg/ml and *in vivo* at 200 ppm in the feed, but were toxic if overdosed (137–141). Dimetridazole was highly effective for treating histomonosis and was rapidly metabolized and eliminated by turkeys with no detectable tissue residue (<0.02 ppm) following 3-day post-administration (142). Enheptin-T (2-amino 5-nitrothiazole) was used at 0.05% in the feed with effective prophylaxis against histomonosis, but average weights of turkeys were suppressed in direct proportion to drug inclusion (143). Nithiazide [1-ethyl-3-(5-nitro-2-thiazolyl) urea] was an effective therapeutic in turkeys when administered at 3-day post-infection and was somewhat better tolerated than enheptin-T (144). Benzimidazole compounds, such as albendazole and fenbendazole, were effective *in vivo* when provided prophylactically and mechanism of action was attributed to damage of heterakid larvae or histomonads residing in the cecal lumen (145).

Research with *H. meleagridis* waned around the 1970s, partly due to effective antihistomonal compounds alleviating disease outbreak, but research increased again in the early 2000s following the removal of effective drugs and feed additives from poultry production in the European Union and the United States which resulted in a re-emergence of disease due to lack of treatment options (6, 67, 146–148). The nitroimidazoles and nitrofurans were banned in the United States in 1987 and 1991, respectively (90, 149). Nitarsone was the last-remaining prophylactic drug for the treatment of histomonosis until the voluntary removal from the US market in late 2015 because of consumer carcinogenic concerns (147, 149–151). Despite occasional success with antihistomonal candidates *in vitro*, subsequent *in vivo* evaluations have failed to conclusively prevent or treat histomonosis (150, 152–156). Boric acid, deoxycholic acid, sodium chlorate, and sodium nitrate are among just a few chemoprophylaxis candidates with antimicrobial or antifungal properties that have been recently tested with *in vitro* evaluation showing significant antihistomonal properties but with no effective prophylaxis *in vivo* (154–156). The antibacterial properties of some candidate antihistomonal compounds are known to impact effectiveness *in vitro*, but histomonads can survive 48 h after destruction of xenic bacterial populations (16, 62, 70, 157). Further complicating the problem, *H. meleagridis* isolates have varied in susceptibility to candidate compounds *in vitro* and *in vivo* (30, 62, 70, 152, 158). Drug resistance was not previously known to occur with *H. meleagridis*; however, some isolates have developed partial resistance to nitarsone and metronidazole, further emphasizing the necessity of new solutions to prevent histomonosis and supporting the likelihood of different populations of protozoa and corresponding drug susceptibility (38, 159, 160). A comparatively reliable compound to replace the previously used dimetridazole and nitarsone drugs is critically needed, but mitigation of histomonosis remains elusive and inconsistent (6, 16). Adaptations likely need to occur for concentration and administration of compounds for *in vivo* protection, but effective *in vitro* evaluation is the initial key step to determining whether to devote resources toward a live animal study (150, 161). *In vitro* methods are useful for initially evaluating candidate chemoprophylactics, but emphasis is placed on *in vivo* evaluation against more than one isolate of *H. meleagridis* before concluding effectiveness.

Paromomycin, an aminoglycoside antibiotic that inhibits protein synthesis, has been effective prophylactically against histomonosis with the target site of action identified as a small subunit rRNA (162–164). Inclusion of paromomycin in the feed at 200 and 400 ppm also reduced *Clostridium perfringens* counts in excreta while reducing *H. meleagridis-*related mortalities under experimental conditions (163). Unfortunately, paromomycin seems limited to prophylactic rather than therapeutic properties, as three commercial turkey flocks in Canada were not successful in reducing mortalities with paromomycin sulfate treatment in the feed (165). Taken together, paromomycin sulfate should be further evaluated as a prophylactic compound for in-feed or in-water administration to prevent *H. meleagridis* infection.

In absence of approved effective drugs or vaccines for histomonosis, the prevailing measure for disease prevention is to minimize exposure to *H. meleagridis*. Worm treatment programs and flock management to prevent *H. gallinarum* and accessory hosts such as earthworms and darkling beetles will help to reduce histomonosis incidence, since histomonads cannot survive for long durations if shed unprotected directly into the environment (15, 16). Limiting exposure to mechanical vectors such as rodents, insects, or contaminated litter is critical to reducing potential contamination. Prompt removal of infected birds and utilization of migration barriers are additional control strategies to prevent rapid horizontal transmission in turkey flocks, while de-worming options would be more appropriate to control histomonosis in chickens based on the differences in bird-to-bird transmission (26, 41, 69).

## Phytochemicals for Prevention of Histomonosis

Phytogenic compounds offer great potential as alternatives to mitigate histomonosis and improve poultry health since the exclusion of antibiotics (166). Herbal products have received much interest for antihistomonal properties, but *in vitro* results are often encouraging while *in vivo* trials yield unsuccessful protection (30, 152, 158). Protophyt^TM^ and Natustat^TM^, plant-derived proprietary combinations of herbal extracts, were successful antihistomonal products *in vitro* but generated only limited success in field trials when provided prophylactically (30, 158, 167–169). Further complicating the search and development of antihistomonal drugs, different monoculture strains of *H. meleagridis* have exhibited varied susceptibilities to natural organic compounds (70). Two proprietary blends of plant extract products containing unspecified amounts of *Capsicum* essential oils exhibited antihistomonal and antibacterial effects after only 48 h *in vitro*; furthermore, mode of action was suggested as cell membrane disruption directly on the histomonads rather than attributed to indirect effects of antibacterial reduction, but *in vivo* studies have not yet been conducted (170). Recently, a dietary supplement (adiCox^SOL^PF) comprised of a proprietary mixture of herbal extracts was effective prophylactically and therapeutically against histomonosis in a turkey breeder flock (171). With increasing demand for organic-raised poultry, naturally derived plant compounds offer a certain attraction as they could potentially be utilized in both organic and traditional production facilities. Plant-based compounds are often relatively cheap to produce, leading to a greater likelihood for industry application (161).

Quinine, an alkaloid obtained from *Cinchona* tree bark, has been successfully utilized to combat malaria (172). Early researchers postulated its potential for treating histomonosis; however, researchers hypothesized that an antihistomonal compound would have to be active more than just locally within the intestines because *H. meleagridis* embeds within the cecal lining and migrates to hepatic tissue (2, 103). Tyzzer (135) observed no reduction in histomonosis following injection of unspecified levels of quinine into the veins or muscles of turkeys. Delaplane and Stuart (173) reported quinine sulfate to be ineffective against *H. meleagridis* infection but did not specify the dose or route of administration. Farmer (174) injected 0.1 ml of 10% quinine iodobismuthate with no apparent protection against histomonosis. Tyzzer and Fabyan (103) suggested that a possible reason for the failure of compounds utilized in human amebic infections to protect poultry from histomonosis could be due to histomonads exhibiting a predominantly flagellated form rather than solely an amoebic form, leading to some products being amebicidal but not antihistomonal. Ensuring delivery of chemoprophylactic candidates directly to the ceca is a challenge, and quinine, although recently shown to be an effective antihistomonal *in vitro*, may not have reached the ceca in sufficient concentration to impair the protozoa when evaluated *in vivo* (175). Previously, chickens recognized the bitter taste of quinine and reduced feed intake of diets containing more than 0.2% quinine, but threshold levels have not been established for turkeys (176). A 0.2% dietary inclusion of quinine was hypothesized to be maximum for turkeys as well; however, the days 0–10 body weight gain in the quinine diets was not different (*p* > 0.05) as compared to the basal diet (175). Turkeys may perceive the bitter taste of quinine differently from chickens and subsequently have higher threshold levels than 0.2%, but the impact to performance at higher inclusion levels is unknown. Other antimalarial compounds such as the herb *Artemisia annua* and plant extracts have been tested against *H. meleagridis* with limited success *in vitro* but no protection was transferred to birds when tested *in vivo* (152, 170).

## Immune Response to *H. meleagridis* Infection

Turkeys and chickens recovered from *H. meleagridis* infection have shown a degree of natural resistance, although both species may retain histomonads sub-clinically and thereby serve as carriers (5, 177). Joyner (178) administered 0.05% dimetridazole in the water to *H. meleagridis-*infected turkeys, and the recovered turkeys were resistant to re-infection which suggested a level of acquired immunity. Protective immunity was observed in birds that recovered from histomonosis and were then subsequently re-infected with *H. meleagridis*, but further attempts with immunization have been inconsistent (18, 102, 103, 177, 179, 180). Sera recovered from immune birds failed to confer robust protection to histomonosis when injected into the peritoneum of naïve poultry that were subsequently challenged intracloacally with *H. meleagridis-*infected liver homogenate (180–182). Passive immunity (via peritoneal injection of antisera) or active immunity (via intramuscular or intraperitoneal injection of lysed clonal *H. meleagridis*) failed to protect against wild-type challenge (183, 184). Turkeys surviving *H. meleagridis* infection have exhibited resistance to re-infection while still maintaining populations of the protozoa within the ceca (182). Humoral immunity does not seem to be the primary component of protective immunity to histomonosis, although antibodies may work in combination with local immunity initiated by leukocytes in the ceca (182).

Clarkson (181) reported that turkeys exhibited decreased albumin and elevated globulin concentrations at 12-day post-infection as compared to the non-challenged controls. Similarly, albumin concentrations greatly decreased by 9-day post-infection in chickens subjected to *H. meleagridis* infection, with normal levels of albumin and globulin fractions restored by 12-day post-infection, suggesting disease recovery (185). The immune barrier in purportedly histomonosis-resistant chickens was suggested to be limited to cecal epithelial tissue as *H. gallinarum* could disrupt and overcome any developed immunity (74). Natural and experimental *H. meleagridis* infection produced antibodies in both chickens and turkeys but transfer of antibodies to naïve birds did not successfully confer protection (180, 184). Subsequently, Clarkson (180) suggested that antibody production alone was not a good indicator of histomonosis recovery or immunity to re-infection. Antibody titers of passively immunized birds were increased compared to pre-immunized groups; however, no protection was induced against intracloacal infection with 3 × 10^5^ *H. meleagridis*, possibly due to the experimental challenge dose not accurately mimicking a natural challenge, antibody levels lower than needed for protection, or more likely, serum antibodies not primarily responsible for protection against *H. meleagridis* infection (184). Immunoglobulin A (IgA) levels have been shown to increase throughout the intestine, while immunoglobulin G (IgG) levels particularly increased in the ceca following infection with an established clonal *H. meleagridis* isolate (186).

Heterophils begin to accumulate around histomonads following initial infection, but the protozoa secrete tissue-degrading enzymes to phagocytose leukocytes (44). Total numbers of heterophils increase throughout the body as *H. meleagridis* migrates to parasitize other tissues; other leukocytes involved include macrophages, giant cells, and plasma cells (44, 64, 119, 187). Once the histomonads invade the cecal submucosa or enter the portal blood, degenerating *H. meleagridis* can be observed within the gut-associated lymphoid tissue (44). Plasma levels of glutamic oxaloacetic transaminase can indicate cellular damage and this enzyme can increase in turkeys with liver and cecal damage from histomonosis (28, 29). CD4+ and CD8α+ T cells have been implicated in the immune response to histomonosis (188–190). Recently, populations of CD4+, CD8α+, and non-CD4+CD8α+ T cells in the liver and spleen of turkeys were induced following administration of attenuated *H. meleagridis* as a putative vaccine and subsequent virulent infection (191). Comparative study of chickens and turkeys indicated that vaccination with a monoxenic, clonal culture of live-attenuated *H. meleagridis* resulted in higher systemic immune response in turkeys as compared to chickens, with increased levels of interferon (IFN)-γ producing CD4+ T cells confirmed in the spleens of infected chickens as compared to turkeys (191). Increased T-helper cell type-1 (Th1) and type-2 (Th2) cytokine responses of IFN-γ and IL-13 occurred in chickens which were co-infected with *H. gallinarum* and *H. meleagridis* (192). Chickens developed a stronger pro-inflammatory innate immune response than turkeys, along with higher antibody levels, with specific increase in the Th2 response in cecal and liver tissues to mitigate infection (188). Despite the extracellular nature of *H. meleagridis* which would be expected to stimulate differentiation of Th2 cells, immune response to this pathogen was suggested to be dominated by Th1 rather than Th2 cells (190–193). Turkeys appeared to have a delayed and uncontrolled immune response as compared to chickens when infected with *H. meleagridis*, allowing greater tissue destruction and ultimately higher mortality in turkeys (194).

## Attempted Vaccination With Attenuated Isolates

Tyzzer (102) evaluated avirulent field strains of *H. meleagridis* for immunization against histomonosis, but inoculation of turkeys was required at a young age and constant re-infection was necessary to maintain a level of effective protection. Partial protection was conferred with an attenuated isolate against subsequent cloacal challenge with a virulent isolate; however, administration of histomonads as an immunization incorporated into *Heterakis* spp. ova and likewise challenged did not satisfactorily confer protection (195, 196). The resulting conclusion was that the low-virulent histomonads were not introduced in sufficient numbers via heterakid ova to successfully initiate immune response to protect against virulent challenge (195, 196). Tyzzer (102, 179) reported attenuation of *H. meleagridis* following repeated passage *in vitro* but attempts with immunization did not produce consistent protection. An isolate repeatedly passaged *in vitro* for 6 years resulted in loss of immunizing ability to chickens and turkeys (196). Further study observed a steady decline of immunizing ability of attenuated histomonads after 730, 766, and 1,000 passages *in vitro* (197). Specifically, passage 1,000 was non-pathogenic and had lost nearly all ability to confer protection to either chickens or turkeys against virulent challenge (197).

Long-term serial passaging *in vitro* places selective pressures on *H. meleagridis* and co-cultured bacterial populations. Freshly obtained field samples of histomonads could not grow in the limited bacterial populations of attenuated culture media; similarly, the attenuated protozoa were unable to survive with the field isolates of cecal bacteria (196). Importantly, *in vitro* attenuation of *H. meleagridis* occurred independently of bacterial populations in culture media (60). *In vitro* growth of *H. meleagridis* Hm-L1 strain at 41.5°C for 9 weeks resulted in low pathogenicity while histomonads stored in liquid nitrogen maintained their original virulence (198, 199). Serial *in vivo* passaging of the Hm-L1 attenuated strain from chicken-to-chicken or turkey-to-turkey restored the strain to original virulence (198, 199). Differences in virulence have been found within *H. meleagridis* isolates obtained from different geographical locations, in addition to varied loss of pathogenicity following repeated passaging (131). Furthermore, subpopulations of serially passaged monocultures originating from the same parental isolate have shown a marked difference in virulence, supporting the idea of genetic mutation through repeated serial passaging *in vitro* (131). Long-term passaging *in vitro* (>290 serial passages) resulted in a phenotype shift toward greater tenacity of histomonad survival at lower temperatures and improved growth rates (37). Gross lesion scoring and histology samples have demonstrated the lowered pathogenicity and reduced ability of attenuated isolates to invade host tissues (200). After 295 serial passages *in vitro*, an avirulent strain of *H. meleagridis* parasitized only the cecal region with no translocation to other tissues in chickens or turkeys, while a virulent strain could be identified in cecal, hepatic, and lung tissues (200).

Vaccination attempts for histomonosis have yielded some success in controlled experimental conditions, but a histomonosis vaccine has not yet been developed for commercial application (91, 101, 164, 183, 194, 201–203). A clonal *in vitro* attenuated strain of *H. meleagridis* administered cloacally as a vaccine at day 14 protected turkeys which were subsequently challenged on day 42 with a virulent strain; in-contact turkeys from the vaccination were also resistant to subsequent infection (183). Furthermore, birds which were administered an attenuated clonal strain as a vaccine were negative for *H. meleagridis* DNA in the liver (183). Oral administration of *in vitro* attenuated *H. meleagridis* to turkeys at day-of-hatch has protected against subsequent wild-type challenge with no adverse effects to performance data during the vaccination phase; the oral route would be a preferable administration route for the poultry industry (91). Under experimental conditions, vaccination of layer chickens with attenuated histomonads prevented a drop in egg production upon virulent challenge and pathological histomonosis lesions were also reduced (203). *In vivo* serial passaging five times in chickens and turkeys did not revert virulence to an *in vitro* attenuated strain, demonstrating stability and safety of attenuated histomonads as vaccine candidates (204). An attenuated clonal strain (passage 295) induced cross-protective immunity in turkeys against subsequent challenge with heterologous virulent isolates; however, vaccination occurred at 1 day of age and a booster vaccination occurred at day 14, with challenge administration at 6 weeks of age (205). Repeated intracloacal passaging of *H. meleagridis* in turkeys produced an isolate of low virulence which was successfully used to induce protection against a virulent strain (206). Candidate vaccination isolates have been shown distinctly attenuated as indicated by lowered mortalities (*p* < 0.05), lowered lesion scores (*p* < 0.05), and similar body weight gain (BWG) (*p* > 0.05) as the non-challenged controls during vaccination phases (101). This information is consistent with previous research indicating attenuation of *H. meleagridis* following repeated *in vitro* passage (102, 131, 179, 196). Importantly, administration of non-clonal vaccination isolates on day 14 has conferred protection against challenge with homologous and heterologous virulent isolates; moreover, these conditions potentially better portrayed the field environment where turkeys are exposed to multiple isolates (101). More research remains necessary for histomonosis vaccine development and to elucidate practical methods for industry application.

## Final Remarks

Biosecurity measures to prevent exposure to *H. meleagridis* or vectors of this protozoa are important to reduce histomonosis incidence due to the absence of vaccines or approved drugs. Proper management practices are critical to reducing disease incidence, as birds experimentally reared in a non-challenged environment do not contract histomonosis. Although separate rearing of poultry (e.g., turkeys raised separately from chickens) can reduce disease incidence by limiting contact between asymptomatic carriers and susceptible hosts, an effective prophylactic or vaccination program is still greatly needed. Pairing *in vitro* and *in vivo* experiments is necessary to ensure effectiveness of candidate antihistomonal compounds.

Despite immunological research advancements, a histomonosis vaccine has not been developed for commercial application (164, 194, 202). Clonal *in vitro* attenuated histomonads have been administered orally or cloacally with efficacious protection in experimental settings against virulent challenge without negative performance impacts; however, evaluations have not yet occurred in field conditions against heterologous, multi-isolate challenges (91, 183, 203). Day-of-hatch oral vaccination with live-attenuated histomonads was previously reported as effective, but a booster vaccination was recommended at day 14 for established protection (91, 205). Recent vaccination experiments demonstrated that day-of-hatch administration of attenuated isolates either orally or cloacally did not protect turkeys against subsequent wild-type challenge (101), contrary to previously reported success with oral vaccination at this age (91, 205). Unfortunately, utilizing live histomonads would be difficult for industry application due to the required intracloacal administration, as well as the additional concerns of attenuation stability and inconsistent protective immunity (6, 9). In practicality, the administration of live-attenuated histomonads on a commercial scale with the current methodologies seems unlikely due to the high cost of cell culture propagation and application complexities, although the benefit to further develop a histomonosis vaccine would be tremendous (5, 202).

The overall review of literature reflects the difficulties in mitigating histomonosis, especially in recent years. Dietary inclusion of antihistomonal compounds such as quinine alone was not encouraging for prevention of *H. meleagridis* infection in turkeys, but vaccination appeared somewhat efficacious when live-attenuated histomonads were administered at day 14 via the cloacal route. Unfortunately, the protection against subsequent wild-type challenge of vaccinated turkeys was neither consistent nor robust throughout the literature. Further research should be conducted with phytochemicals as these compounds may offer a natural remedy for histomonosis that could be both economical for the industry and acceptable to the consumer. Vaccination should be pursued further, especially to elucidate the administration route, dose, and age of bird. Taken together, this information is encouraging for immunity to histomonosis, but the administration of a vaccine and possible requirement for booster vaccination with the live-attenuated method is more experimentally interesting rather than industry applicable.

## Author Contributions

LB and CV developed the conceptualization and wrote the first draft of the manuscript. LB, VP-G, and GT-I conceptualized and created the figures. LB, GT-I, BG, BH, and CV participated in the design, analysis, presentation, and writing of the manuscript. All authors contributed to the article and approved the submitted version.

## Funding

This research was supported in part by the funds provided by the USDA-NIFA Sustainable Agriculture Systems, Grant No. 2019-69012-29905. Title of the project: Empowering US Broiler Production for Transformation and Sustainability USDA-NIFA (Sustainable Agriculture Systems): No. 2019-69012-29905.

## Conflict of Interest

The authors declare that the research was conducted in the absence of any commercial or financial relationships that could be construed as a potential conflict of interest.

## Publisher's Note

All claims expressed in this article are solely those of the authors and do not necessarily represent those of their affiliated organizations, or those of the publisher, the editors and the reviewers. Any product that may be evaluated in this article, or claim that may be made by its manufacturer, is not guaranteed or endorsed by the publisher.
